# Functional Analysis of an Intronic FBN1 Pathogenic Gene Variant in a Family With Marfan Syndrome

**DOI:** 10.3389/fgene.2022.857095

**Published:** 2022-04-25

**Authors:** Kui Hu, Yun Wan, Fu-Tsuen Lee, Jinmiao Chen, Hao Wang, Haonan Qu, Tao Chen, Wang Lu, Zhenwei Jiang, Lufang Gao, Xiaojuan Ji, Liqun Sun, Daokang Xiang

**Affiliations:** ^1^ Department of Cardiovascular Surgery, Guizhou Provincial People’s Hospital, Guiyang, China; ^2^ Department of Endocrinology, Guizhou Provincial People’s Hospital, Guiyang, China; ^3^ Department of Physiology, Temerty Faculty of Medicine, University of Toronto, Toronto, ON, Canada; ^4^ Division of Cardiology, Department of Pediatrics, The Hospital for Sick Children, University of Toronto, Toronto, ON, Canada; ^5^ Department of Cardiovascular Surgery, Zhongshan Hospital, Fudan University, Shanghai, China; ^6^ Institute of Precision Medicine, The Ninth People’s Hospital, Shanghai Jiao Tong University School of Medicine, Shanghai, China; ^7^ Department of Thoracic and Cardiovascular Surgery, The Third People’s Hospital of Mianyang City, Mianyang, China; ^8^ Department of Ultrasound, Chongqing General Hospital, University of Chinese Academy of Sciences, Chongqing, China

**Keywords:** Marfan syndrome, intronic variant, fibrillin 1, RNA splicing, genetic analysis

## Abstract

Marfan syndrome (MFS) is an autosomal dominant connective tissue disorder that canonically affects the ocular, skeletal, and cardiovascular system, in which aortic tear and rupture is the leading cause of death for MFS patients. Genetically, MFS is primarily associated with fibrillin-1 (FBN1) pathogenic variants. However, the disease-causing variant in approximately 10% of patients cannot be identified, partly due to some cryptic mutations that may be missed using routine exonic sequencing, such as non-coding intronic variants that affects the RNA splicing process. We present a 32-year female with typical MFS systemic presentation that reached to a clinical diagnosis according to the revised Ghent nosology. We performed whole-exome sequencing (WES) but the report failed to identify known causal variants when analyzing the exonic sequence. However, further investigation on the exon/intron boundaries of the WES report revealed a candidate intronic variant of the fibrillin 1 (FBN1) gene (c.248-3 C>G) that predicted to affect the RNA splicing process. We conducted minigene splicing analyses and demonstrated that the c.248-3 C>G variant abolished the canonical splicing site of intron 3, leading to activation of two cryptic splicing sites and causing insertion (c.248-1_248-2insAG and c.248-1_248-282ins). Our study not only characterizes an intronic variant to the mutational spectrum of the FBN1 gene in MFS and its aberrant effect on splicing, but highlights the importance to not neglect the exon/intron boundaries when reporting and assessing WES results. We point out the need of conducting functional analysis to verify the pathogenicity of intronic mutation, and the opportunity to re-consider the standard diagnostic approaches in cases of clinically diagnosed MFS with normal or variant of unknown significance genetic results.

## 1 Introduction

Marfan syndrome (MFS) is an autosomal dominant connective tissue disorder with variable penetrance at an estimated incidence of 2–3 in 10,000 individuals (Groth et al., 2015). Typical pathological manifestations of MFS involve the skin, skeleton, ocular system, and cardiovascular systems, from which aortic dissection or rupture resulting from progressive aorta dilation is the leading cause of mortality. However, prophylactic aortic surgery with comprehensive medical treatment has substantially improved the survival of MFS patients (Milewicz et al., 2021). Pathogenetic variants in the fibrillin 1(FBN1) gene encoding fibrillin-1—an extracellular matrix protein have been identified as the primary disease-associated gene in MFS (Maslen et al., 1991). Up to a quarter of *FBN1* pathogenetic variants are *de novo* while a clear family history is apparent in the majority of MFS probands (Chiu et al., 2014). Currently, the clinical diagnosis of MFS is based on a set of manifestations from the revised Ghent nosology, including assessment of systemic features and measurements of the thoracic aorta (Loeys et al., 2010). In situations of clinical uncertainty or preclinical diagnosis, molecular genetic testing is an integral part of the diagnostic and differential diagnosis process, as well as genetic counseling (Milewicz et al., 2021). Moreover, a definitive genetic diagnosis facilitates the screening of potentially affected relatives and future offspring. However, the genetic cause in about 10% of patients with typical MFS phenotypes and clinical diagnosis according to Ghent nosology cannot be identified (Zeigler et al., 2021). This is partially due to cryptic pathogenic variants outside the conventional exonic area of FBN1 that has been previously described, such as rare pathogenic variants in the middle of introns that lead to splicing of intron sequences into FBN1 transcript or haploinsufficiency, which is due to nonsense-mediated decay of the mutant transcript (Guo et al., 2008; Torrado et al., 2018; Wu et al., 2021).

In this study, we report a clinically diagnosed MFS female with typical clinical MFS characteristics. Standard genetic testing was performed to identify the causal variant for MFS but our clinical report of the exonic sequence failed to confirm the disease-associated mutation. Further investigation of the exon/intron boundaries identified an intronic variant of the FBN1 gene (c.248-3 C>G), and computer prediction and functional study using minigene splicing assays verified the pathogenicity of this cryptic mutation. Furthermore, we screened the proband’s family members and identified two preclinical relativities with the same pathogenic variant who are now under medical treatment with follow-up.

## 2 Materials and Methods

### 2.1 Patient Clinical Information

A 32-year-old female was admitted to our emergency department for acute chest pain. She was 162 cm tall, but had marfanoid systemic manifestations. The patient mentioned that her grandmother, two of her uncles, her mother, and one of her cousins passed away suddenly in their twenties or thirties. In addition, one of her cousins underwent cardiovascular surgery for acute aortic dissection in his twenties. Therefore, MFS was suspected in this patient.

### 2.2 Clinical Examination for Marfan Syndrome

Based on first physical presentation of the patient, an emergency computed tomography angiography (CTA), transthoracic echocardiography (TTE), and electrocardiogram (ECG) were performed for an MFS diagnosis. Blood and urine specimens were collected and tested. Clinical MFS characteristics were evaluated: the cardiovascular system (e.g., aortic root dilatation, aneurysm, and dissection, mitral valve prolapse), ocular system (e.g., ectopia lentis, myopia), skeletal system (e.g., arachnodactyly, scoliosis, pectus excavatum, hindfoot deformity, dolichocephaly, enophthalmos, malar hypoplasia, retrognathia), and skin striae. The scoring of MFS systemic features was calculated according to the revised Ghent nosology (Loeys et al., 2010).

### 2.3 Genetic Analysis

As per manufacturer’s protocol, a QIAamp DNA Blood Mini Kit (Qiagen GmbH, Hilden, Germany) was used to extract and purify 1 μg of DNA from 200 μl sampled blood. The DNA libraries were constructed using a polymerase change reaction (PCR)-free method. Next-generation sequencing was applied for mutation screening. The platforms for whole-exome sequencing (NanoWES Human Exome, Berry Genomics Corporation, Beijing, China) were performed on a Illumina NovaSeq 6000 (Illumina, San Diego, USA). Single-nucleotide polymorphisms (SNPs), insertions, or deletions (InDels), splicing (SPIDEX, dbscSNV, spliceAI and NetGene2) were determined by bioinformatics analysis. The pathogenicity of variants was evaluated based on the American College of Medical Genetics and Genomics (ACMG) standards and guidelines (Richards et al., 2015). The sequencing reads were aligned to the human reference genome (hg38/GRCh38).

### 2.4 Minigene Analysis

*In vitro* minigene splicing assay was performed for the splicing variant as previously described (Xiong et al., 2021). Generally, the wild-type (WT) and mutant-type (MT) forms of the minigene regions, encompassing exons 3–5, intron 3, and partial intron 4 of FBN1, were amplified from genomic DNA of the proband, using the following primer pairs: 5′- AAG​CTT​GGT​ACC​GAG​CTC​GGA​TCC​ACC​CAA​TGT​CTG​TGG​ATC​ACG​TTA​TAA​T-3′ and 5′- ATG​AGA​CAA​GAA​TTA​TGA​CTC​ACT​TGC​CCA​AAC​CCC​C-3′, 5′-GAG​TCA​TAA​TTC​TTG​TCT​CAT​ATG​GTT​ACT​CAA​GGC​A-3′ and 5′-TTA​AAC​GGG​CCC​TCT​AGA​CTC​GAG​GTT​GTC​CAC​AGT​GAG​TCC​CTA​TGT​ATC​C-3′. The amplified products were cloned into a pMini-CopGFP vector (Beijing Hitrobio Biotechnology Co., Ltd.) double digested at the restriction sites BamHI and XhoI using a ClonExpress II One Step Cloning Kit (Vazyme, Nanjing, China). Sanger sequencing was used to verify WT and MT minigene plasmids to be selected for further transfection. As per protocol previously described (Xiong et al., 2021), human embryonic kidney 293T (HEK293T) cells were cultured and incubated for recombinant plasmid transient transfection with Lipofectamine 2000 (Invitrogen, Carlsbad, CA, United States). After 48 h, total RNA was extracted from cells with TRIzol reagent (Cowin Biotech Co., Jiangsu, China). Reverse transcription-PCR (RT-PCR) was performed with the primer pair: 5′-GGC​TAA​CTA​GAG​AAC​CCA​CTG​CTT​A-3′ and 5′-GTT​GTC​CAC​AGT​GAG​TCC​CTA​TGT​A-3′. Agarose gel electrophoresis and Sanger sequencing were used to analyze PCR fragments and to determine gene isoforms, respectively. Thereafter, the Expasy-tranlate tool (https://web.expasy.org/translate/) was used to translate the nucleotide sequence to the protein sequence to analyze the effect of mutation on translation.

## 3 Results

### 3.1 Proband Clinical Presentation and Management

**FIGURE 1:**
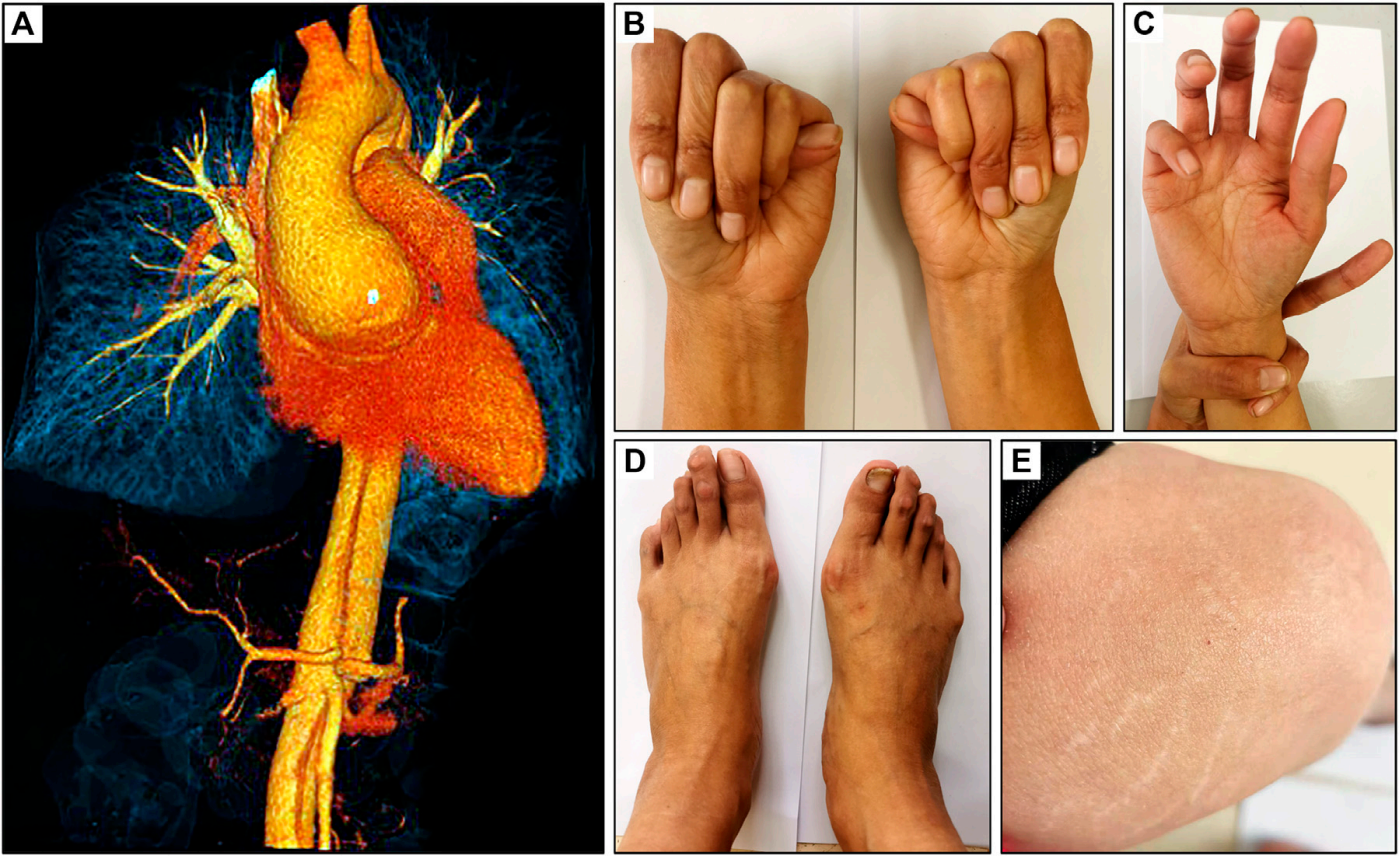
Clinical presentation of a 32-year-old female with MFS. **(A)** CT scan revealed an aortic root dilatation and Debakey I dissection, **(B)** positive thumb sign, **(C)** positive wrist sign, **(D)** plain pes planus and **(E)** skin striae.

Emergency CTA showed dilatation of the ascending aorta (6.2 cm) and a Debakey I dissection which affected the ascending aorta, aortic arch and branches, descending aorta, abdominal aorta, celiac trunk, superior mesenteric artery, and left renal artery (Figure 1A). TTE examination demonstrated dilatation of the aortic root and severe regurgitation of the aortic valve. According to the revised Ghent criteria, the patient’s systemic score was 7/20 points based on the presence of positive wrist and thumb signs, pectus carinatum deformity, plain pes planus, and skin striae (Figures 1B–E), which reached the threshold for an MFS clinical diagnosis.

Emergency surgical repair was indicated for the acute Debakey I dissection involved in the patient. During the surgical procedure, extracorporeal circulation *via* cardiopulmonary bypass was established and the patient underwent the Bentall procedure, aortic arch replacement, descending aortic endovascular stent-graft implantation. The patient had an uneventful recovery, was prescribed a β-adrenergic receptor blocker, and scheduled for regular follow-up.

### 3.2 Proband FBN1 Genetic Analysis

Standard exonic sequence reporting for the suspicion of MFS failed to identify a causal FBN1 pathogenic variant for the proband. However, further investigation of the exon/intron boundaries sites identified a candidate intronic mutation (NM_000138.4: c.248-3C>G), which was considered as a variant of unknown significance (PM2+PP1+PP3+PP4) according to ACMG standards and guidelines. The pathogenic variant (c.248-3C>G) in the FBN1 gene was predicted to abrogate the intron 3 canonical acceptor splice site by using SPIDEX, dbscSNV, spliceAI and NetGene2 and Beef Data & Genomics Programme.

### 3.3 Family FBN1 Genetic Screening and Clinical Assessments

**FIGURE 2:**
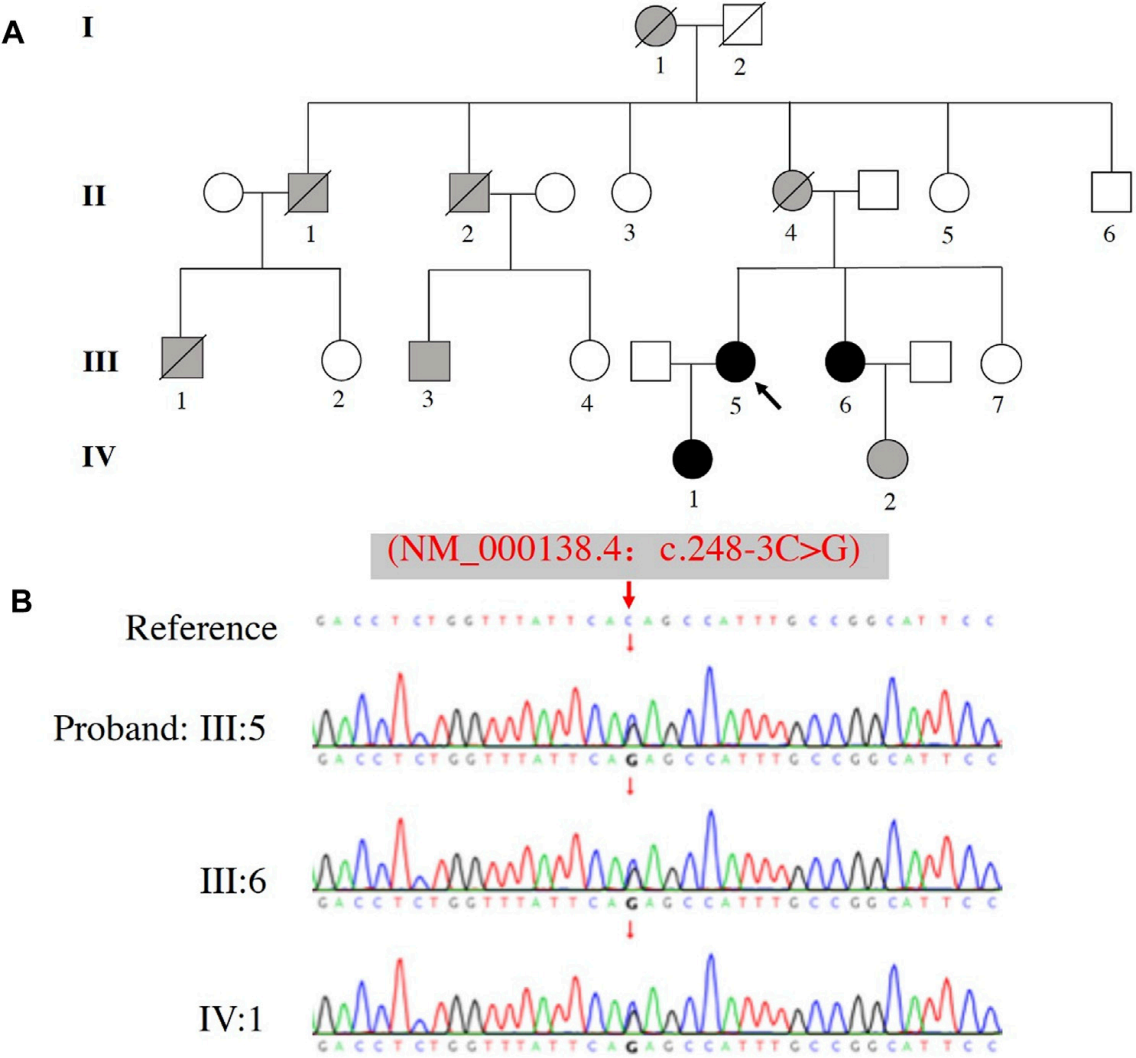
Pedigree and sequence analysis of a family with a history of MFS. **(A)** Family pedigree, black arrow indicates the proband (III: 5), blank filling indicates not affected, black filling indicates affected and genetically confirmed, gray filling indicates affected but not genetically tested. Lines through the shapes indicates deceased members. **(B)** The variant c.248-3C>G of FBN1 was identified in the proband (III: 5), her sister (III: 6), and daughter (IV: 1).

The identified FBN1 pathogenic gene variant was confirmed in all family members that were genetically tested by Sanger sequencing, which included the proband (III:5), her sister (III: 6), and daughter (IV: 1) (Figure 2A). All 3 cases carried the same variant c.248-3C>G FBN1 mutation (Figure 2B).

Clinical assessments of the related family members are shown in Table 1. Five cases (I:1, II:1, II:2, II:4 and III:1) died around their thirties (range: 28–36 years-old) without autopsy, but all members had marfanoid skeletal features based on the family history. Fortunately, III:3 (first cousin) and III:5 (proband) were both treated by emergency cardiovascular surgery and survived. Assessment of three other family members (III:6, IV:1 and IV:2) revealed they also carried some MFS systemic features, including long fingers, chest deformity, and a mildly dilated aortic root. β-adrenergic receptor blocker was prescribed to these three individuals to reduce the growth rate of aortic dilatation and were scheduled to receive a TTE follow-up assessment every 6–12 months.

**TABLE 1:** Clinical features of the affected family members.

Pedigree ID	Age (year)	Treatment/Surgery/major complication/cause of death	Clinical presentation
I:1	36	Sudden death (no autopsy)	Marfanoid skeletal features
II:1	33	Sudden death (no autopsy)	Marfanoid skeletal features
II:2	32	Sudden death (no autopsy)	Marfanoid skeletal features
II:4	34	Sudden death (no autopsy)	Marfanoid skeletal features
III:1	28	Sudden death (no autopsy)	Marfanoid skeletal features
III:3	28	Debakey I dissection; Bentall surgery, aortic arch replacement, descending aortic endovascular stent-graft implantation (Alive)	Severe aortic valve insufficiency, Wrist and thumb signs, Pectus carinatum deformity, Plain pes planus, Skin striae, Myopia
III:5	32	Debakey I dissection; Bentall surgery, aortic arch replacement, descending aortic endovascular stent-graft implantation (Alive)	Severe aortic valve insufficiency, Wrist and thumb signs, Pectus carinatum deformity, Plain pes planus, Skin striae
III:6	31	β-adrenergic receptor blocker treatment (Alive)	Mild aortic root dilation, Mild aortic valve insufficiency, Skin striae
IV:1	8	β-adrenergic receptor blocker treatment (Alive)	Mild aortic root dilation, Wrist and thumb signs, Pectus carinatum deformity, Skin striae, Mild scoliosis
IV:2	6	β-adrenergic receptor blocker treatment (Alive)	Mild aortic root dilation, Wrist and thumb signs, Pectus carinatum deformity, Skin striae, Mild scoliosis

### 3.4 The Pathogenic FBN1 c.248-3C>G Gene Variant

**FIGURE 3:**
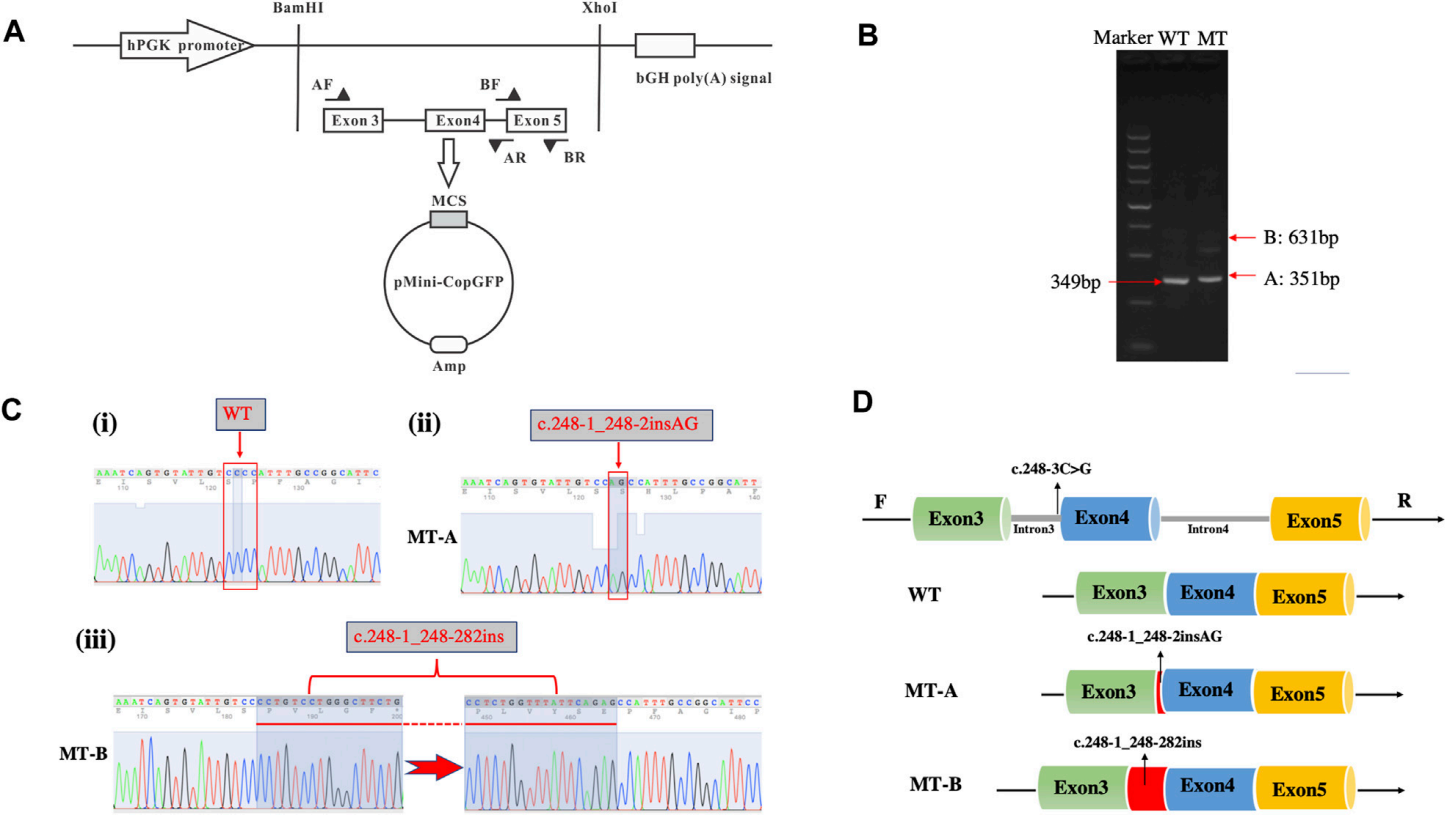
Minigene assay for FBN1 c.248-3C>G variant and schematic diagram of the splicing pattern. **(A)** Minigene trapping vetor construct; **(B)** Gel electrophoresis of RT-PCR revealed a single band for wild-type and two bands for mutant-type; **(C)** minigene product sequencing demonstrated that the wild-type minigene formed normal mRNA (i), but the c.248-3C>G substitution of FBN1 caused a splicing abnormality, which abrogate the intron 3 canonical splice site and lead to activating two cryptic sites in exon 4, resulting in a 2 bp insertion (ii) and 282 bp insertion (iii); **(D)** the schematic diagram of splicing pattern of WT, MT-A and MT-B.

Figure 3A illustrates the minigene trapping vetor construct and electrophoresis of the RT-PCR products displayed a single band for WT and two bands for MT (Figure 3B). Sanger sequencing revealed a normal splicing isoform for WT (Figure 3C i), and aberrant splicing for MT, resulting in the insertion of 2 nucleotides (lower band) or of 282 nucleotides (upper band) in exon 4 (Figures 3Cii,iii). Analysis of the minigene splicing assay suggested that the c.248-3C>G substitution can abrogate the intron 3 canonical acceptor splice site and lead to activating two cryptic sites in exon 4, which predicted to cause insertion (c.248-1_248-2insAG and c.248-1_248-282ins) (Figure 3D).

### 3.5 Bioinformatic Analysis

**FIGURE 4:**
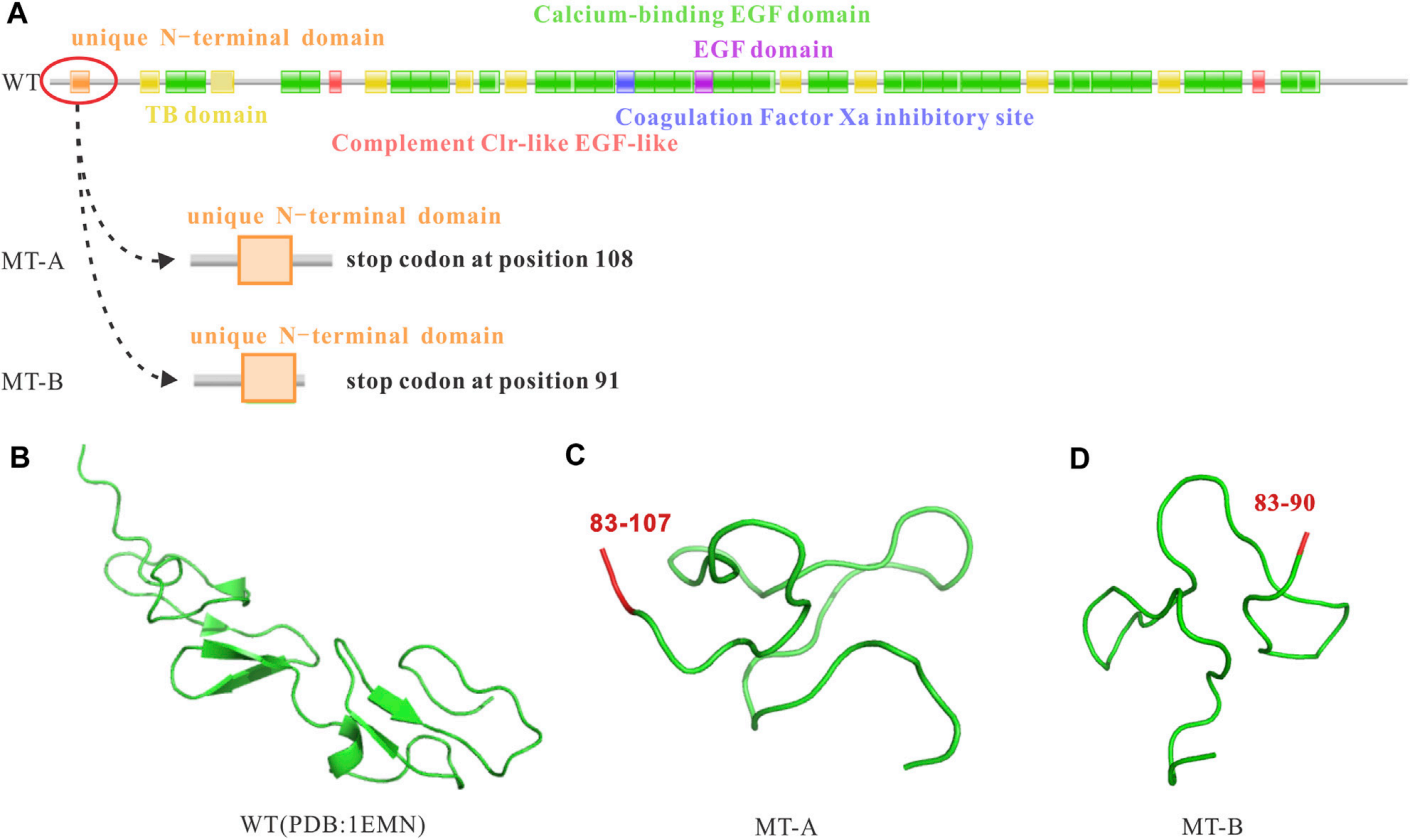
Impact of the FBN1 c.248-3C>G variant on the reading frame resulting in premature stop codons and truncated proteins. **(A)** WT reading frame, MT-A causing a premature stop codon at position 108, and MT-B causing a premature stop codon at position 91; **(B)** WT protein; **(C)** MT-A resulting in a truncated protein; **(D)** MT-B resulting in a truncated protein.

To explore whether the random 2 bp or 282 bp insertion were associated with downstream FBN1 dysregulation, we analyzed the coding potential of the three sequences. Two inserts were first aligned back into the WT allele and deciphered abide by the central dogma. However, translation of the nucleotide to protein sequence revealed, not only did the 2 bp insertion induce a coding-frame shift leading premature translational termination, but also the 282 bp variant sequence incurred several stop codons based on the open reading frame of the WT which interrupted the translational process and resulted in truncated proteins (Figure 4).

## 4 Discussion

In this study, we report the identification of an intronic pathogenic FBN1 c.248-3C>G gene variant found in family with MFS, which includes the proband and 2 genetically tested members. Based on the revised Gent nosology, systemic scoring of all 3 members achieved a clinical MFS diagnosis. Initially, standard clinical genetic reporting of the exonic sequence failed to identify a disease-associated variant. However, further investigation of the exon/intron boundaries identified a candidate variant that was predicted to affect splicing. Although the candidate was assessed as a variant of unknown significance according to the ACGM standards and guidelines, *in vitro* minigene splicing assay demonstrated that the intronic mutation abrogates the canonical splice site of intron 3 and activates two cryptic sites, resulting in 2 bp or 282 bp insertion respectively on exon 4. These insertions were predicted to interrupt the translational process, resulting in truncated proteins. Our study not only adds a new intronic pathogenic variant to the mutational spectrum of the FBN1 gene in MFS but also highlights the importance of including exon/intron boundaries for clinical genetic reporting and the need to conduct functional analyses to verify the pathogenicity of intronic mutations as previously described (Wu et al., 2021).

Marfan syndrome is a connective tissue disorder inherited in an autosomal dominant manner with variable penetrance (Pyeritz, 2016). Multiple organ systems are frequently affected and without appropriate and timely treatment, patients have an increased risk of mortality (Reed E. Pyeritz, 2019). The major cause of death in MFS patients is cardiovascular complications, particularly progressive dilatation of the proximal aorta, which can lead to aortic dissection or fatal rupture (Vanem et al., 2018). It wasn’t until 1991 that the mutation in FBN1 gene was identified as the genetic locus responsible for MFS (Maslen et al., 1991). Since then, great progress has been made on the understanding of the pathogenesis, diagnosis, and treatment as well as follow-up for MFS individuals. FBN1 is located on the long arm of chromosome 15 and has 65 coding exons that encodes for fibrillin-1. Fibrillin-1 is a protein macromolecule that polymerizes into microfibrils which are fibers that provide load bearing structural support in all connective tissues. Almost 2000 pathogenetic variants in FBN1 predisposing to MFS have been identified to date and are distributed throughout the gene (Pinard et al., 2019).

Despite significant progress made in understanding the genetic and molecular basis of MFS, it has been reported only 12% of FBN1 mutations causing Marfan syndrome appear more than once in unrelated individuals, an observation that proposes the need for expanded identification of cryptic mutations for a definitive MFS diagnosis (Hiratzka et al., 2010). There are many different types of mutations in the FBN1 gene, but nonsense and missense mutations are more frequently observed in MFS patients. In approximately 10%–15% of all reported mutations, a majority are associated with premature stop codons from small insertions, deletions, or duplications. Another 10%–15% of these pathogenic variants are composed of different variations of splicing errors, most of which have been reported to affect canonical splice sites at the exon/intron boundaries (Robinson et al., 2006). The cryptic pathogenetic rare variants such as those located in the middle of introns that lead to the splicing of intron sequences into the FBN1 transcript, and haploinsufficiency due to small insertions, frameshift in translation, deletions and stop codons, somatic mosaicism, as well as nonsense-mediated decay of the mutated transcript, should be investigated as standard protocols for genetic diagnostic testing will not detect these mutations (Guo et al., 2008; Arnaud et al., 2021). Furthermore, functional studies are necessary to confirm these cryptic mutations. Although entire gene deletions are rare, larger rearrangements have been reported in a minority of MFS patients, including both deletions and insertions (Schrijver et al., 2002; Faivre et al., 2007; Faivre et al., 2009; Hilhorst-Hofstee et al., 2011). Given the increasingly large spectrum of mutations found in FBN1 and remaining undiscovered etiologies, genetic screening of the FBN1 gene has been also suggested to include supplemental techniques for detecting large/deletions and deep-intronic mutations using cDNA analysis and whole-genome sequencing in addition to standard testing for exonic mutations in cases of clinically diagnosed MFS with normal or variant of unknown significance genetic results. (Yang et al., 2018; Gillis et al., 2014). In the present study, analysis in the exonic area failed to identify the disease-causing variant. While further investigation on exon/intron boundaries identified a potential intronic mutation in FBN1 causal for MFS, an *in vitro* cell study confirmed that the intronic mutation affected the splicing process. Translational analysis verified that both aberrant insertions resulted from the intronic mutation will lead to translation failure through stop codon incursion.

In the past decades, extensive progression has been made for the diagnosis and treatment of MFS, including prophylactic surgery, medical treatment, and regular follow up, which has greatly improved the life-expectancy in patients with MFS. For adults with an enlarged aorta or aneurysm, surgical repair is indicated when the diameter reaches to 5.0 cm (Hiratzka et al., 2010; Erbel et al., 2014). Meanwhile, pharmacological treatment has been worthwhile strategy to reduce the enlargement of aortic dilatation, thus avoiding cardiac surgery and its associated complications if the aorta is not significantly enlarged. This includes medications affecting myocardial inotropy and heart rate, or targeting signaling pathways that have been implicated in the pathogeneses of MFS (Jennifer et al., 1994; Hiratzka et al., 2010). The 2010 American Heart Association (AHA)/American College of Cardiology (ACC) Thoracic Aortic Disease guidelines recommends that patients with MFS and aortic aneurysms take β-adrenergic receptor blocker therapy to reduce the rate of aortic dilatation unless contraindicated (Hiratzka et al., 2010). Angiotensin receptor blockers have also demonstrated to be beneficial in reducing aortic dilatation in randomized controlled trials (Hofmann Bowman et al., 2019).

The importance for a definitive genetic diagnosis cannot be underestimated. For example, the definitive genetic diagnosis of MFS facilitates the screening of family members who may have inherited pathogenic variant but have not manifested obvious multi-systemic abnormalities, and aids in genetic counseling for affected parents preparing for pregnancy (Chen et al., 2021) and comprehensive prenatal screening for pregnant women if *de novo* MFS is suspected *in utero*, albeit rare (Wang et al., 2020). Furthermore, genetic testing may also differentiate MFS from other related disorders that share similar systemic abnormalities arising from variants different from FBN1 (Halper, 2014). In our experience, children and adolescents with preclinical manifestations of MFS are frequently only seen by chance or discovered through genetic screening of the family’s proband due to progressive MFS symptoms that calls for clinical evaluation. Increasing our understanding behind the different types of mutations involved in MFS can lead to a timely definitive genetic diagnosis and subsequent screening, evaluation, and if needed, pharmacological therapy to reduce aortic dilatation or prophylactic surgery to avoid aortic dissection or fatal rupture. In this study, the clinical diagnosis of the proband and identification of a pathogenic variant, which was also subsequently identified in 2 family members, are now treated with β-adrenergic receptor blockers and scheduled for follow-up appointments.

## 5 Conclusion

This study reports the identification of an intronic pathogenic FBN1 c.248-3C>G gene variant found in a family with MFS, which was found to have an aberrant effect on splicing. Our findings highlight the need to not neglect the exon/intron boundaries of whole exome sequencing for clinical genetic reporting, and the need of conducting functional analysis to verify the pathogenicity of intronic mutations. Furthermore, this calls for the reconsideration of standard diagnostic approaches in cases of clinically diagnosed MFS with normal or variant of unknown significance genetic results. A definitive genetic diagnosis is not only conducive to screening family members to find patients with atypical clinical symptoms, especially children and adolescents, but also to genetic counseling for parents preparing for pregnancy and prenatal screening for pregnant women.

## Data Availability

The datasets for this article are not publicly available due to concerns regarding participant/patient anonymity. Requests to access the datasets should be directed to the corresponding authors.
